# Supplementary material for: Steroid Metabolomic Signature in Term and Preterm Infants
Source: Biomolecules. 2024 Feb 17;14(2):235. doi: 10.3390/biom14020235 (PMC10887377; doi:10.3390/biom14020235)
Supplement: Supplementary file 1 [file biomolecules-14-00235-s001.zip › Suppl Table S1_GI.pdf]

Suppl. Table S1. List of the urinary steroid metabolites and their origin analyzed in term, preterm and early preterm infants.

Abbreviation	Urinary Steroid Metabolite	Origin of Urinary Steroid
THS	5β-Pregnane-3α,17,21-triol-20-one	11-deoxycortisol
15ß,17OH-P5o	5-Pregnene-3β,15β,17-triol-20-one	17-hydroxypregnenolone
P5-tetrol-15ß	5-Pregnene-3β,15β,17,20α-tetrol	17-hydroxypregnenolone
17OHPo	5β-Pregnane-3α,17-diol-20-one (17–OHPregnanolone)	17-hydroxyprogesterone
17OHPo-5α	5α-Pregnane-3α,17-diol-20-one	17-hydroxyprogesterone
15ß,17OHPo	5β-Pregnan-3α,15β,17-triol-20-one	17-hydroxyprogesterone
PT	5β-Pregnane-3α,17, 20α-triol (Pregnanetriol)	17-hydroxyprogesterone
THA	5β-Pregnane-3α,21-diol-11,20-dione	Corticosterone
THF	5β-Pregnane-3α,11β,17,21-tetrol-20-one (Tetrahydro-Cortisol)	cortisol
F	4-Pregnene-11β,17,21-triol-3,20-dione (Cortisol)	cortisol
6ß-OH-F	4-Pregnene-6β,11β,17,21-tetrol-3,20-dione	cortisol
20α-DHF	4-Pregnene-11β,17,20α,21-tetrol-3-one	cortisol
11-O-An	5α-androstane-3α-ol-11,17-dione	11-hydroxyandrostenedione
11-OH-An	5α-Androstane-3α,11β-diol-17-one	11-hydroxyandrostenedione
THE	5β-Pregnane-3α,17,21-triol-11,20-dione (Tetrahydro-Cortisone)	cortisone
α-Cl	5β-Pregnane-3α,17,20α,21-tetrol-11-one (α-Cortolone)	cortisone
6α-OH-THE	5β-Pregnane-3α,6α,17,21-tetrol-11,20-dione	cortisone
1ß-OH-THE	5β-Pregnane-1β,3α,17,21-tetrol-11,20-dione	cortisone
ß-Cl	5β-Pregnane-3α,17,20β,21-tetrol-11-one (β-Cortolone)	cortisone
6α-OH-α-Cl	5β-Pregnane-3α,6α,17,20α,21-pentol-11-one	cortisone
6α-OH-ß-Cl	5β-Pregnane-3α,6β,17,20β,21-pentol-11-one	cortisone
1ß-OH-ß-Cl	5β-Pregnane-1β,3α,17,20β,21-pentol-11-one	cortisone
A5-3ß,17α	5-Androstene-3β,17α-diol	DHEA
DHEA	5-Androstene-3β-ol-17-one (Dehydroepiandrosterone)	DHEA
Adiol	5-Androstene-3β,17β-diol	DHEA
16α-OH-DHEA	5-Androstene-3β,16α-diol-17-one	DHEA
16ß-OH-DHEA	5-Androstene-3β,16β-diol-17-one	DHEA
15ß,16αOH-DHEA	5-Androstene-3β,15β,16α-triol-17-one	DHEA
16-O-A5D	5-Androstene-3β,17β-diol-16-one	DHEA
A5T16α	5-Androstene-3β,16α,17β-triol (Androstenetriol-16α)	DHEA
A5T-16ß	5-Androstene-3β,16β,17β-triol	DHEA
16α,18OH-DHEA	5-Androstene-3β,16α,18-triol-17-one	DHEA
A5-3ß16α17ß18-tetrol	5-androstene-3β,16α,17β,18-tetrole	DHEA
A5-3ß15ß16α17ß-tetrol	5-androstene-3β,15β,16α,17β-tetrole	DHEA
16OHP5o	5-Pregnene-3β,16α-diol-20-one	Pregnenolone
21OHP5o	5-Pregnene-3β,21-diol-20-one	Pregnenolone
P5-3ß20α21-triol	5-Pregnene-3β,20α,21-triol	Pregnenolone
P5-tetrol	5-Pregnene-3β,16α,20α,21-tetrole	Pregnenolone
