# Supplementary material for: Steroid Metabolomic Signature in Term and Preterm Infants
Source: Biomolecules. 2024 Feb 17;14(2):235. doi: 10.3390/biom14020235 (PMC10887377; doi:10.3390/biom14020235)
Supplement: Supplementary file 1 [file biomolecules-14-00235-s001.zip › Suppl Table S2.pdf]

Suppl. Table S2A. Graphical representation of the daily urinary steroid metabolome in term infants (> 37 weeks gestational age). Steroids are shown divided into six excretion rate ranges (median, µg/kg/d) illustrating their relative contribution to the total circulating steroid pool.

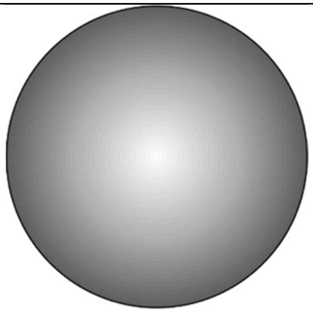<br>> 1000	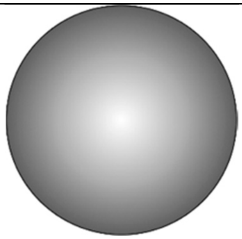<br>500-1000	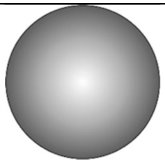<br>200-500	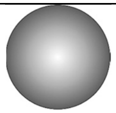<br>100-200	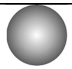<br>10-99	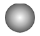<br><10
A5-3ß16α17ß18-tetrol	P5-3ß20α21-triol	16α-OH-DHEA<br><br>16OHP5o	6α-OH-ß-Cl<br><br>16ß-OH-DHEA<br>15ß,16αOH-DHEA<br>16-O-A5D<br>A5T16α<br>16α,18OH-DHEA<br>A5-3ß15ß16α17ß-tetrol<br><br>P5-tetrol-15ß	F<br><br>THE<br>6α-OH-THE<br>ß-Cl<br>6α-OH-α-Cl<br><br>15ß,17OHP5o<br><br>21OHP5o<br>P5-tetrol	THF<br>6ß-OH-F<br><br>1ß-OH-THE<br>1ß-OH-ß-Cl<br>THA<br><br>11-O-An<br>11-OH-An<br><br>A5-3b,17a<br>DHEA<br>Adiol<br><br>17OHPo<br>17OHPo-5α<br>15b,17OHPo<br>PT

Suppl. Table S2B. Graphical representation of the daily urinary steroid metabolome in preterm infants (30-36 weeks gestational age). Steroids are shown divided into six excretion rate ranges (median, µg/kg/d) illustrating their relative contribution to the total circulating steroid pool. * different contribution compared to terms

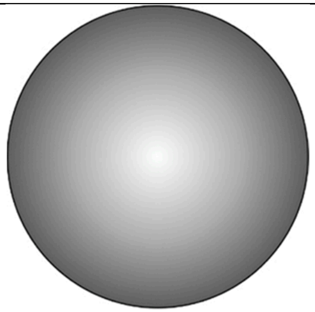<br>> 1000	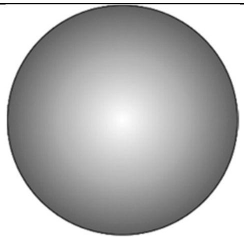<br>500-1000	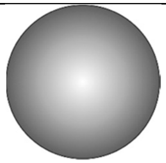<br>200-500	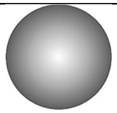<br>100-200	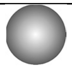<br>10-99	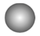<br><10
	P5-3ß20α21-triol<br>16α-OH-DHEA*<br>A5-3ß16α17ß18-tetrol*	16OHP5o<br>16-O-A5D*<br>A5T16a*<br>P5-tetrol-15ß*	6α-OH-ß-Cl<br><br>16ß-OH-DHEA<br>16α,18OH-DHEA<br>A5-3ß15ß16α17ß-tetrol<br>15ß,17OHP5o*	THE<br>6α-OH-THE<br>α-Cl<br>ß-Cl<br>6ß-OH-α-Cl<br><br>15ß,16αOH-DHEA*<br><br>21OHP5o<br>P5-tetrol	F*<br>6ß-OH-F<br><br>1ß-OH-THE<br>1ß-OH-ß-Cl<br>THA<br><br>THS*<br>11-O-An<br>11-OH-An<br>A5-3ß,17α<br>DHEA<br>Adiol<br>A5T16ß*<br>17OHPo<br>17OHPo-5α<br>15ß,17OHPo<br>PT

Suppl. Table S2C. Graphical representation of the daily urinary steroid metabolome in early preterm infants (<30 weeks gestational age). Steroids are shown divided into six excretion rate ranges (median, µg/kg/d) illustrating their relative contribution to the total circulating steroid pool. * different contribution compared to terms

> 1000	500-1000	200-500	100-200	10-99	<10
A5-3ß16α17ß18-tetrol	16α-OH-DHEA*	16-O-A5D*<br>A5T16α*<br>16α,18OH-DHEA*<br>A5-3ß15ß16α17ß-tetrol*<br>16OHP5o*<br>P5-tetrol-15b*<br>P5-3ß20α21-triol*	6α-OH-ß-Cl<br><br>16ß-OH-DHEA<br>15ß,17OHP5o*<br>15ß,16αOH-DHEA<br>P5-tetrol*	F<br>6ß-OH-F*<br>THE<br>α-Cl<br>ß-Cl<br>6α-OH-α-Cl<br><br><br>21OHP5o	6α-OH-THE*<br>1ß-OH-ß-Cl<br>20α-DHF*<br>THA*<br>THS*<br><br>11-O-An<br>11-OH-An<br><br>A5-3ß,17α<br>DHEA<br>A5T-16ß*<br>17OHPo<br>17OHPo-5α<br>15ß,17OHPo<br>PT
